# Auxetic Photonic Patterns with Ultrasensitive Mechanochromism

**DOI:** 10.1002/advs.202304022

**Published:** 2023-11-09

**Authors:** Hwan‐Young Lee, Minbon Gu, Jeonghee Hwang, Hyerim Hwang, Young‐Seok Kim, Su Yeon Lee, Shin‐Hyun Kim

**Affiliations:** ^1^ Department of Chemical and Biomolecular Engineering Korea Advanced Institute of Science and Technology (KAIST) Daejeon 34141 Republic of Korea; ^2^ Division of Advanced Materials Korea Research Institute of Chemical Technology (KRICT) Daejeon 34114 Republic of Korea; ^3^ Advanced Materials and Chemical Engineering University of Science and Technology (UST) Daejeon 34113 Republic of Korea; ^4^ Department of Chemical Engineering and Materials Science Ewha Womans University Seoul 03760 Republic of Korea; ^5^ Korea Electronics Technology Institute (KETI) Seongnam Gyeonggi‐do 13509 Republic of Korea

**Keywords:** auxetic patterns, mechanochromism, photonic bandgap, photonic crystals, structure colors

## Abstract

Photonic crystals with mechanochromic properties are currently under intensive study to provide intuitive colorimetric detection of strains for various applications. However, the sensitivity of color change to strain is intrinsically limited, as the degree of deformation determines the wavelength shift. To overcome this limitation, auxetic photonic patterns that exhibit ultra‐sensitive mechanochromism are designed. These patterns have a regular arrangement of cuts that expand to accommodate the strain, while the skeletal framework undergoes torsional deformation. Elastic photonic crystals composed of a non‐close‐packed array of colloidal particles are embedded in the cut area of the auxetic patterns. As the cut area amplifies the strains, the elastic photonic crystals show significant color change even for small total strains. The degree of local‐strain amplification, or sensitivity of color change, is controllable by adjusting the width of cuts in the auxetic framework. In this work, a maximum sensitivity of up to 60 nm/% is achieved, which is 20 times higher than bulk films. It is believed that the auxetic photonic patterns with ultra‐sensitive mechanochromism will provide new opportunities for the pragmatic use of mechanochromic materials in various fields, including structural health monitoring.

## Graphical abstract

Auxetic photonic patterns are designed to provide ultra‐sensitive mechanochromism. As the strains are locally amplified in the cut areas of the auxetic patterns, elastic photonic crystals in the cuts show significant color change even for small total strains. The sensitivity is as high as 60 nm/%, which is 20 times improved from bulk films, enabling intuitive colorimetric monitoring of strains.

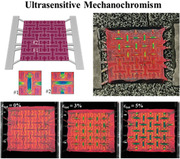

## 1 Introduction

Panther chameleons have a unique ability to change the color of their skin by manipulating iridophores that contain non‐close‐packed regular arrays of guanine nanocrystals.^[^ ^1^, ^2^, ^3^ ^]^ These regular arrays diffract a specific wavelength of light, resulting in structural colors, which is a common principle behind the structural coloration by photonic crystals.^[^ ^4^, ^5^, ^6^, ^7^, ^8^, ^9^, ^10^, ^11^, ^12^, ^13^, ^14^, ^15^ ^]^ The chameleons can control the expansion and contraction of the arrays in the iridophores to dynamically tune the colors.^[^ ^1^, ^2^, ^3^ ^]^ Inspired by this mechanism, mechanochromic photonic structures have been artificially created using colloidal crystals. One method involves filling interstitial voids of close‐packed colloidal crystals with elastomeric materials to make composites.^[^ ^16^, ^17^, ^18^, ^19^, ^20^ ^]^ Another method involves removing colloidal particles from the composites to create inverse‐opal structures.^[^ ^21^ ^]^ Additionally, arrays of core‐shell particles can be compressed at high temperature to produce mechanochromic films made up of a non‐close‐packed array of cores in an elastic matrix of fused shell.^[^ ^22^, ^23^, ^24^, ^25^ ^]^ Alternatively, mechanochromic materials can be produced by dispersing colloidal particles in a photocurable elastomer precursor where the particles spontaneously crystallize due to repulsive interparticle potential, which is then captured by photopolymerization of the precursor.^[^ ^26^, ^27^, ^28^, ^29^, ^30^ ^]^

The mechanochromic properties of materials are promising for a wide range of applications, including wearable displays, anti‐counterfeiting patches, and colorimetric sensors.^[^ ^31^, ^32^, ^33^, ^34^ ^]^ Elastic materials can be used to make color changes highly reversible, while low‐modulus materials can increase sensitivity to stress.^[^ ^16^, ^21^, ^22^, ^30^ ^]^ However, even for purely elastic deformation with a Poisson's ratio of 0.5, large strains are still required to induce significant color change. This is because the degree of lattice deformation directly determines the shift of the diffraction wavelength;^[^ ^22^, ^24^, ^30^ ^]^ the sensitivity of the wavelength shift against strain for previous mechanochromic materials is summarized in Table S1 (Supporting Information). Despite this, most applications require high sensitivity to strain. For example, mechanochromic materials need to detect very low strains to monitor the structural health of materials such as concrete, steel, and composites. Displays also require color changes across the full visible range with small displacement that electronic devices can provide. Therefore, it remains as an important challenge to make the mechanochromic property to be highly sensitive to strain for practical use of mechanochromic materials.

In this study, we have developed innovative designs for mechanochromic materials by combining elastic photonic structures with auxetic patterns. Auxetic patterns are a type of mechanical metamaterial that exhibit negative in‐plane Poisson's ratios due to a specific hierarchical cut design.^[^ ^35^, ^36^, ^37^, ^38^, ^39^, ^40^, ^41^, ^42^, ^43^, ^44^, ^45^ ^]^ We have utilized strain localization in the cuts of the auxetic patterns to achieve ultra‐sensitive mechanochromism. A colloidal ink is inserted into the cuts of auxetic patterns and then solidified. The low modulus of the elastic photonic structure, in comparison to the auxetic framework, does not significantly affect the deformation behavior of the framework with the void cuts. Importantly, the local strain in the photonic structure is amplified, causing significant color changes even for small overall strain. The width of the cuts controls the degree of strain localization and amplification. By using a narrow cut, the maximum sensitivity achieved is 60 nm/%, which is 20 times greater than the bulk‐film sensitivity of 3 nm/%. The sensitivity amplification is mainly achieved by the auxetic patterns, which is proved by the sensitivity to local strains in the cuts same as the bulk film. These photonic auxetic patterns are highly sensitive in visualizing strains with color change, making them promising as colorimetric strain sensors for structural health monitoring.

## 2 Results and Discussion

### 2.1 Production of Auxetic Photonic Patterns

Auxetic materials that exhibit a negative Poisson's ratio possess a unique mechanical property of in‐plane expansion along all directions when stretched in one direction.^[^ ^35^, ^36^, ^37^, ^38^, ^39^, ^40^, ^41^, ^42^, ^43^, ^44^, ^45^ ^]^ This peculiar behavior is predominantly driven by the expansion of the cut areas, while the skeletal material experiences minimal deformation; hinge parts of the skeletal experience torsional deformation rather than tensile deformation.^[^ ^38^, ^39^, ^40^ ^]^ This strain localization in the cut areas of the anomalous mechanical patterns is advantageous for designing ultra‐sensitive mechanochromic patterns with normal elastic photonic materials, which have not been utilized previously.

**Figure 1:**
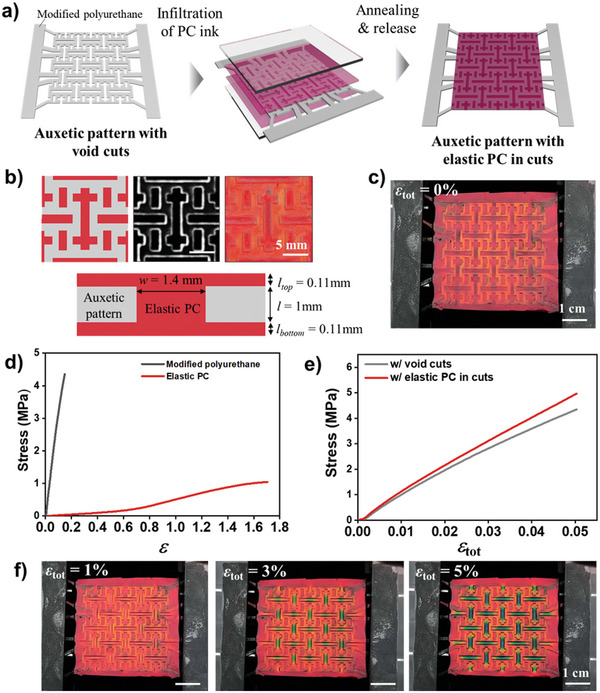
Auxetic photonic patterns. a) Schematics for the production of auxetic photonic pattern. Elastic photonic crystals (PCs) are embedded in the cuts of auxetic framework made of modified polyurethane and coated on top and bottom sides by sandwiching the framework in the presence of photonic inks with a pair of two parallel glasses and photocuring the ink. b) Unit‐cell design (top left), optical microscope (OM) images of the auxetic framework (top middle) and photonic pattern (top right), and schematic cross‐section of the photonic pattern (bottom). The dimensions are indicated in the bottom schematics. c) Photograph of an auxetic photonic pattern. d) Strain–stress curves for the bulk films of modified polyurethane and elastic PC. e) Strain–stress curves for the auxetic framework and photonic pattern f) Series of photographs of the auxetic photonic patterns with the strain of 1% (left), 3% (middle), and 5% (right). The elastic PCs show a blueshift of the reflection color along with the strain.

To create the auxetic framework, we first generate a master mold using 3D printing. We duplicate the master mold with polydimethylsiloxane (PDMS), which is then used as a mold to prepare the auxetic framework. The auxetic framework is made by photopolymerization of a photocurable mixture of urethane acrylate and isobornyl acrylate in a weight ratio of 3:1 with the PDMS mold, as shown in Figure S1 (Supporting Information). To create the elastic photonic structures, we use a photocurable dispersion of monodisperse silica particles with an average diameter of 228 nm in poly(ethylene glycol) phenyl ether acrylate (PEGPEA) at a volume fraction of 0.40. Additionally, carbon black nanoparticles are included in the dispersion at a concentration of 0.03 w/w% to enhance color saturation by reducing random scattering.^[^ ^46^ ^]^ The silica particles in PEGPEA have a repulsive potential that causes them to form a non‐close‐packed array, which is then captured in an elastomeric matrix through the photopolymerization of PEGPEA.^[^ ^17^ ^]^ The auxetic framework is sandwiched between two glass slides in the presence of the silica‐PEGPEA dispersion, as illustrated in **Figure** **1**a. The dispersion fills the void cuts of the framework and forms thin layers on the top and bottom of the framework. After thermal annealing and ultraviolet (UV) irradiation, the freestanding pattern is released from the glass slides.

The design of the auxetic pattern comprises two expandable cuts of vertical double‐crosses and horizontal lines and one rotational deformation‐indicating cut of short vertical lines, as depicted in the top panels of Figure 1b. The width and height of the cuts are fixed at 1.4 and 1 mm, respectively, as illustrated in the bottom panel of Figure 1b. The thin layers of photonic structures on the top and bottom of the auxetic framework are ≈0.11 mm thick, which improve the adhesion between the photonic structures and the auxetic framework. As the auxetic framework is made of polyurethane, it is transparent and durable. When the elastic photonic structures are introduced into the framework, the auxetic pattern becomes red due to the selective reflection at the central wavelength of 676 nm, as shown in Figure 1c and Figure S2 (Supporting Information). The auxetic pattern has a dimension of 4.1 × 4.1 cm^2^.

The polymerized PEGPEA containing silica particles at the volume fraction of 0.40 is highly stretchable and elastic. To characterize the mechanical property of the photonic structure, the same silica‐PEGPEA dispersion is cast into a bulk film with a thickness of 1 mm by infiltrating the dispersion into a gap between two parallel slide glasses and photopolymerizing it. The films show a linear response of stress to tensile strain up to the strain of 50%, at which the modulus is measured as 0.28 MPa, as shown in Figure 1d. For the strains >50%, the modulus increases to 0.98 MPa, which is attributed to the contact between silica particles by the deformation. The films break at the strain of 170%. We also measure the modulus of the framework materials. A bulk film is prepared with a mixture of urethane acrylate and isobornyl acrylate in a weight ratio of 3:1 by following the same film casting protocol as photonic films. The films show a modulus of 27.07 MPa and break at the strain of 14.9%, as shown in Figure 1d. That is, the framework material has a modulus two orders of magnitude higher than the elastic photonic material, which is beneficial for conserving the deformation behavior of the framework in the photonic structure‐embedded patterns.

The mechanical properties of the framework are also characterized using the tensile test. The modulus is measured to be 8.63 MPa, which is much lower than that of the framework material, as shown in Figure 1e. This is expected since the framework with void cuts experiences torsional deformation with minimal tensile deformation. The photonic patterns, which are elastic photonic structure‐embedded frameworks, exhibit a modulus of 9.87 MPa, which is slightly higher than that of the framework. Since the elastic photonic structure has a modulus as low as 0.28 MPa, it does not provide strong resistance against the deformation. Therefore, the mechanical response of the photonic patterns is not significantly different from that of the framework.

The photonic patterns exhibit significant color changes from red to orange, green, and blue in the cut areas as the strain increases from 0 to 1%, 3%, and 5%, respectively, as shown in Figure 1f and Movie S1 (Supporting Information). In contrast, the color change from red to blue in the bulk film requires a strain of at least 45%, as shown in Figure S3 and Movie S3 (Supporting Information). This indicates that the sensitivity of the color change to strain is roughly ten times higher for the auxetic photonic patterns due to the localization and amplification of strain in the cut areas. The color change is highly reversible since the deformation is highly elastic, as demonstrated in Movie S2 (Supporting Information). When the framework is made from urethane acrylate in the absence of isobornyl acrylate, the relatively low modulus of the framework results in less localized strain in the cut areas, leading to a lower degree of color change, as shown in Figure S4 (Supporting Information).

### 2.2 Quantification of Mechanochromism

**Figure 2:**
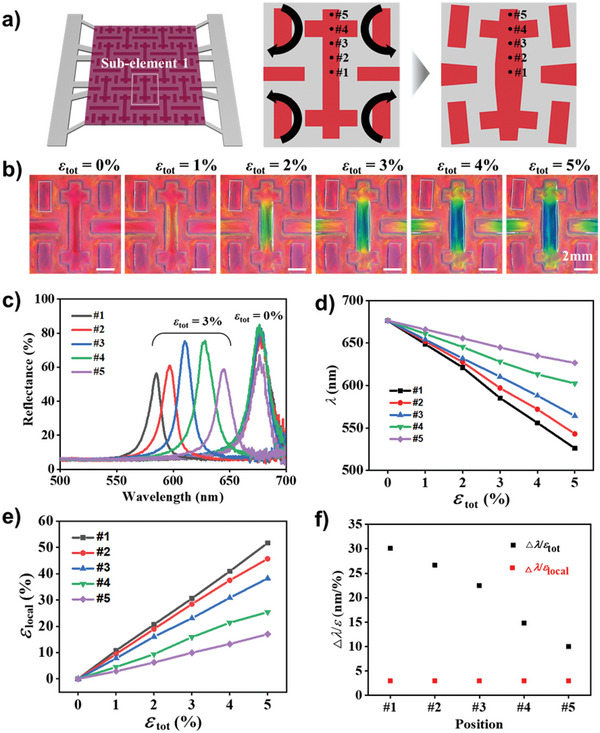
Sensitivity of mechanochromism in double‐crosses element. a) Schematics showing the cut element of double‐crosses at strain‐free state (middle) and stretched state (right). b) Series of OM images showing the uneven color change in the double‐cross along with strain as denoted. One of the four short rectangular cuts is highlighted with a box, which rotates, without color change, along with strain. c) Reflectance spectra taken from five different locations, denoted in (a), at total strains of 0% and 3%. d) Reflectance peak wavelengths and e) local strain at five different locations as a function of total strain. f) Sensitivities of the reflectance peak shift to total strain (black squares) and local strain (red squares) at the five different locations.

To quantify the strain amplification and color change, two cut elements, consisting of vertical double‐crosses and horizontal lines, are characterized. The cut elements of the double‐crosses are inserted perpendicular to the tensile direction, as illustrated in **Figure** **2**a. As the strain increases, the width of the vertical line of the double‐crosses also increases, with the maximum width observed at the center, as shown in Figure 2b and Movie S4 (Supporting Information). The uneven change in the width is accompanied by the rotation of the four rectangular cuts around the double‐crosses. The deformation in the double‐crosses causes a blueshift of the colors, which is also maximized at the center, as expected. As the double‐cross is symmetric along the centerline, the blueshift at five different local positions in the top half of the double‐cross is analyzed, as denoted in Figure 2a. The reflectance spectra are acquired from the local positions for total strains ranging from 0 to 5%, as shown in Figure S5 (Supporting Information). The reflectance peaks are observed at 676 nm regardless of the positions, which shifted to 585 nm at the center (position #1) and 645 nm near the end (position #5) for a total strain of 3%, for example, as shown in Figure 2c. The reflectivity decreases as the strain increases due to the reduction in the refractive index contrast between the particle‐rich and particle‐poor layers in the composite.^[^ ^17^ ^]^

The strain‐dependent peak wavelengths for the five spots are presented in Figure 2d. A nearly linear correlation between strain and wavelength is observed for all five spots. The sensitivity, defined as the degree of blueshift divided by the strain, is 30 nm/% at the center and 10 nm/% near the end, where a rough linear correlation exists between position and sensitivity. The sensitivity at the center is ten times higher than that of the bulk film which exhibits the sensitivity of ≈3 nm/%, as shown in Figure S3c (Supporting Information). The enhanced sensitivities originate from the localized and amplified strains in the cuts. The local strains at the five spots are directly measured from the microscope images in Figure 2b. As the length of the double‐crosses remains almost unchanged, the change in width normalized by the initial width is measured as the local strain. The local strains at the five spots linearly increase along with the total strain, as shown in Figure 2e. The local strain at the center is 10.3 times magnified relative to the total strain; the local strain is 51.7% for a total strain of 5%. This amplification of the local strain is almost the same as that observed for the auxetic framework with void cuts, as shown in Figure S6 (Supporting Information). This further confirms that the embedding of the elastic photonic structure insignificantly alters the mechanical responses of the auxetic framework for strains <5%.

The sensitivities at the five spots are summarized in Figure 2f, and it is observed that the sensitivity is higher where the local strain is more amplified. The local sensitivity, which is the blueshift of the peak position divided by the local strain, remains constant at ≈3 nm/% for all five positions, which is consistent with the bulk film in Figure S3 (Supporting Information). Therefore, there is no degradation in the mechanochromic performance of the elastic photonic structures embedded in the cuts of auxetic patterns. For the bulk film, a linear correlation between the strain and the blueshift is conserved up to a strain of 50%. In the auxetic patterns, the maximum local strain is 50% on the center, which is within the range of linear behavior. Therefore, the sensitivity of the bulk film is conserved for the cuts in the auxetic patterns. It is noteworthy that the short rectangular cuts maintain their color and reflectance spectrum while slightly rotating, as shown in Figure 2b and Figure S7 (Supporting Information). This can be used as a reference to quantify the deviation in the color for strain analysis.

**Figure 3:**
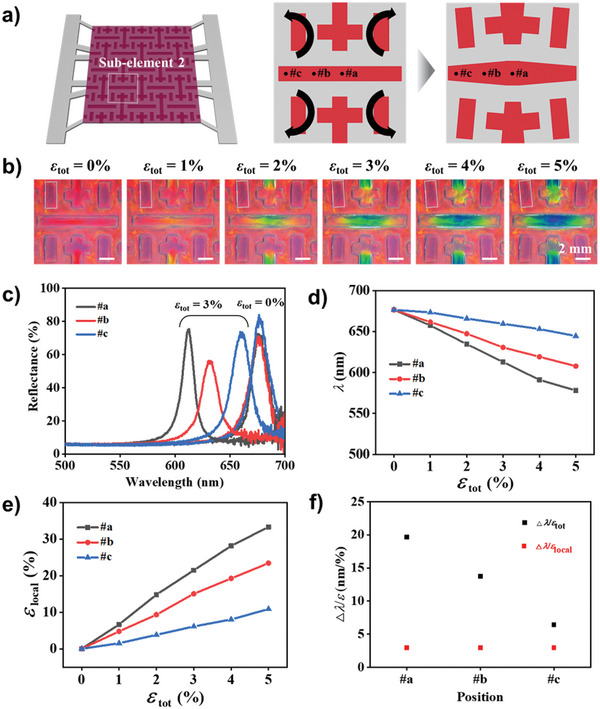
Sensitivity of mechanochromism in horizontal‐line element. a) Schematics showing the cut element of the horizontal line at strain‐free state (middle) and stretched state (right). b) Series of OM images showing the uneven color change in the horizontal line along with strain as denoted. One of the four short rectangular cuts is highlighted with a box, which rotates, without color change, along with strain. c) Reflectance spectra taken from three different locations, denoted in (a), at total strains of 0% and 3%. d) Reflectance peak wavelengths and e) local strain at three different locations as a function of total strain. f) Sensitivities of the reflectance peak shift to total strain (black squares) and local strain (red squares) at the three different locations.

The cut elements of the horizontal lines were inserted to be parallel to the tensile direction, as illustrated in **Figure** **3**a. As the strain increases, so does the width of the lines, as shown in Figure 3b and Movie S5 (Supporting Information). Similar to the double‐crosses, the horizontal lines also experience uneven widening, with the center showing the largest widening and blueshift. For instance, the center (position #a) shows a blueshift of the reflectance peak from 676 to 612 nm for 3% strain, whereas the near‐edge (position #c) shows a shift to 659 nm, as shown in Figure 3c. The spectra for the full range of strain are presented in Figure S8 (Supporting Information). The strain‐dependent blueshifts at three different positions are summarized in Figure 3d. The large blueshifts compared to the bulk film arise from the amplified local strains, as shown in Figure 3e. When the local sensitivity is taken into account, the bulk sensitivity of ≈3 nm/% is conserved, as depicted in Figure 3f. The sensitivity in the horizontal lines is 20 nm/% at the center, which is lower than the 30 nm/% observed in the center of double‐crosses. This difference in sensitivity can be attributed to the slightly lower degree of local strain amplification in the horizontal lines, as indicated by the consistent local sensitivity. Since the horizontal lines are parallel to the tensile direction, they experience a slightly weaker widening compared to the perpendicular double‐crosses. This difference in local strain between the two cut elements is also observed in the auxetic framework with void cuts, as shown in Figure S6c (Supporting Information).

The auxetic photonic pattern exhibits consistent color changes and recovery throughout multiple cycles of stretching and relaxation, without hysteresis, as depicted in Figure S9 (Supporting Information). The maximum strain applied to the pattern is limited to 5% due to the local strain reaching ≈50% with a ten times amplification, which represents the boundary for a linear response of the bulk film. The pattern remains free from plastic deformation or failure up to a strain of 12%, with failure occurring at the interfaces between photonic materials and the auxetic pattern when the strain exceeds 13%, as shown in Figure S10 (Supporting Information). This high stability is achieved by producing a thin film of photonic materials that envelops the entire auxetic pattern, as depicted in Figure 1b. While the auxetic photonic patterns can withstand total strains of up to 12%, strains beyond 5% result in a nonlinear response and reduced reflectivity due to the inherent limitations of the photonic materials.

### 2.3 Control over the Sensitivity

**Figure 4:**
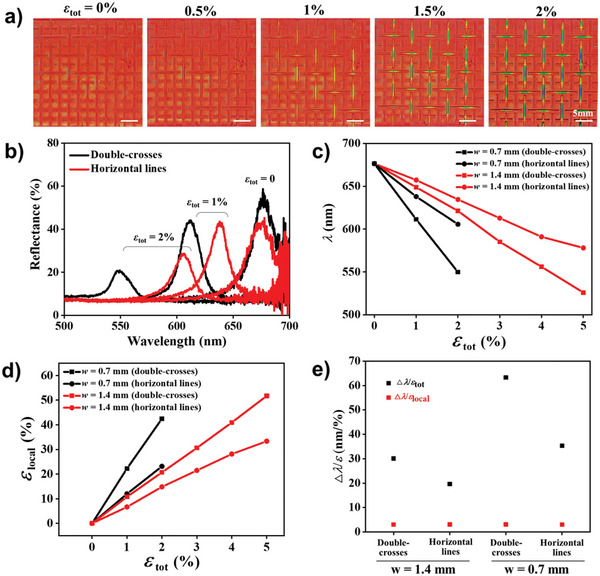
Enhancement of sensitivity. a) Series of photographs of an auxetic photonic pattern with a cut width of 0.7 mm at various total strains, as denoted. b) Reflectance spectra taken from the centers of double‐cross and horizontal line at the strains of 0, 1%, and 2%. c) Reflectance peak wavelengths and d) local strain at the centers of double‐cross and horizontal line in the auxetic patterns with cut widths of 0.7 and 1.4 mm, respectively. e) Sensitivities of the reflectance peak shift to total strain (black squares) and local strain (red squares) for the centers of double‐cross and horizontal line in the auxetic patterns with cut widths of 0.7 mm and 1.4 mm.

The sensitivity of mechanochromism is determined by the amplification of local strain in the cut areas. To enhance the sensitivity, the cut width is reduced from 1.4 to 0.7 mm while maintaining the auxetic pattern design, as depicted in **Figure** **4**a. The narrower cuts lead to higher amplification as the strain is more localized. The color on the center of the double‐crosses shifts from red to yellow at 1% strain, green at 1.5%, and blue at 2%, as illustrated in Figure 4a and Movie S6 (Supporting Information). The reflectance peak at the center of double‐crosses shifts from 676 nm at strain‐free state to 611 nm at 1% strain and 549 nm at 2% strain, as shown in Figure 4b. The reflectance peak at the center of horizontal lines is 638 nm at 1% strain and 605 nm at 2% strain. The peak shifts for the auxetic patterns containing cuts with 0.7 mm width are much stronger than the patterns with 1.4 mm width, as presented in Figure 4c.

The larger shifts originate from more amplified local strains in the pattern with 0.7 mm cut‐width than 1.4 mm cut‐width, as demonstrated in Figure 4d. Consequently, the sensitivity on the center of the 0.7‐mm‐width double‐crosses is as high as 63 nm/%, and that on the horizontal lines is 35 nm/%, which are approximately two times larger than the 1.4‐mm‐width cuts, as summarized in Figure 4e. The local sensitivity in the 0.7‐mm‐width cuts remains unchanged at ≈3 nm/% as the local strain is in the range of linear response of the bulk film up to the total strain of 2%. It is noteworthy that the narrower cuts have a more limited range of colorimetric measurement of the strain, although they have a higher sensitivity; the auxetic photonic patterns support total strains up to 4% without failure, as shown in Figure S11 (Supporting Information). Therefore, it is necessary to set the cut width in the auxetic patterns by considering the strain range and precision of measurement for the specific target applications.

### 2.4 Colorimetric Measurement of Structural Deformation

**Figure 5:**
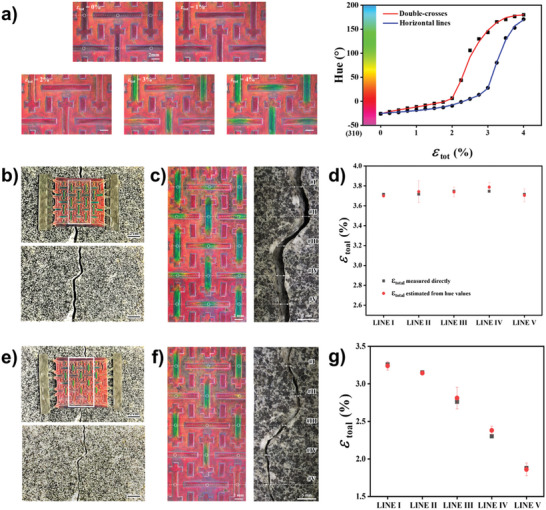
Intuitive monitoring of structural health. a) Series of images showing strain‐dependent color change of the auxetic photonic pattern (left panels) and standard curves for strain measurement from hue values at central regions of double‐crosses and horizontal lines. b) The auxetic photonic pattern attached on a brick, where a gap with almost constant width is created by separating two blocks perpendicular to the crack after the attachment (top panel) and the brick from which the pattern is removed (bottom panel). c) Images showing the color change of the pattern on the brick (left panel) and the gap width (right panel) at five different positions. d) Strain estimated from the hue values and directly measured from the gap width. e–g) The same set to (b–d) for the asymmetric gap in the brick created by slight tilting of two blocks.

The photonic auxetic patterns display a high sensitivity of color change to strain, making them potentially useful as colorimetric strain sensors in various fields, including the intuitive monitoring of the structural health of materials. To quantify strain using color change, we first prepare standard curves to establish the relationship between strain and hue values, as depicted in **Figure** **5**a. Hue values are extracted from the central regions of double‐crosses and horizontal lines of the auxetic patterns under controlled strains and are subsequently averaged. Full images used for the curves are included in Figure S12 (Supporting Information). There is a one‐to‐one correspondence between strain and hue value in the strain range of 0–4%. With the use of the standard curves, we can estimate strains from hue values.

To demonstrate the practical use as colorimetric sensors for structural health monitoring, we affix two paddles of the patterns onto a brick that had been pre‐broken into two blocks and recombined. We then create a gap along the crack by separating the two blocks perpendicular to the crack, as shown in the top panel of Figure 5b. The auxetic pattern displays a blueshift of colors in the double‐crosses and horizontal lines when strained, as depicted in the left panel of Figure 5c. The strain is estimated from the hue values at the central regions of the double‐crosses and horizontal lines, based on the standard curves, at five different positions. Three color‐changing components, comprising either two double‐crosses and one horizontal line or one double‐cross and two horizontal lines, are included in each position, from which strains are measured and averaged. As the two blocks are separated without tilting, the strains measured at five different positions are consistent, at 3.7%, as shown in Figure 5d. To confirm the reliability of the estimation, the auxetic pattern is removed from the brick while maintaining the two blocks in place, as shown in the bottom panel of Figure 5b. The gaps between the two blocks are directly measured at the five different positions from the image in the right panel of Figure 5c. The strains are then calculated from the gaps by taking into account the distance between two paddles of the auxetic pattern, as shown in Figure 5d. The strains estimated from the hue values and those directly measured coincide with negligible deviation.

To investigate the ability of the photonic auxetic patterns to measure the distribution of strain, we create an asymmetric gap by separating two blocks such that there is a gap on the top and no gap on the bottom. The auxetic pattern displays a blueshift in the color‐changing components, with the degree of the blueshift gradually decreasing along the direction from the top to the bottom, as shown in the top panel of Figure 5e and the left panel of Figure 5f. The strains are measured from three components and averaged at five different positions, as shown in Figure 5g. The strain at position #I is 3.2%, which decreases to 1.8% at position #V. The strains are also separately calculated from the direct measurement of gaps in the right panel of Figure 5f. The position‐dependent strains directly measured are consistent with those estimated from the hue values. This indicates that the photonic auxetic patterns can provide the spatial distribution of strain from the color map. Although various sophisticated techniques exist for precise structural health monitoring, inexpensive disposable patches often rely on basic rulers. However, reading these rulers becomes challenging when they are applied to inaccessible areas of structures. In such cases, colorimetric monitoring offers an advantage by enabling direct analysis using a camera on drones, even when the images are out of focus or blurred, rendering the use of rulers ineffective.

## 3 Conclusion

In this study, we create auxetic photonic patterns that exhibit ultrasensitive mechanochromism. We design an auxetic framework with a negative Poisson's ratio to localize and amplify strain in cut areas. We embed elastic photonic crystals of non‐close‐packed colloidal arrays in the cut area of this framework. As the modulus of the elastic photonic crystals is two orders of magnitude smaller than the modified polyurethane in the framework, the mechanical response of the framework is well‐preserved in the composite auxetic patterns. Consequently, the elastic photonic crystals in the cut areas display significant color change due to amplified local strain, even for small total strain. By adjusting the cut width of the auxetic patterns, we can control sensitivity. We achieve a maximum sensitivity of 60 nm/% in this study, which is 20 times higher than that of the bulk films. With this enhanced sensitivity, we can detect strains less than 1% with the naked eye. Moreover, it is possible to quantitatively analyze the infinitesimal strain change by analyzing the reflectance spectrum. As sensitivity and strain range are in trade‐off, the cut width should be adjusted by considering the required sensitivity and strain range for target applications. It is possible to modify the size of silica particles to regulate the initial wavelength of the stopband, allowing for the transition from infrared to visible or visible to ultraviolet. Additionally, different designs of auxetic patterns can be utilized, wherein the width of the patterns is appropriately adjusted based on the desired applications. Although we utilize macroscopic auxetic patterns created through 3D printing for strain measurement at the centimeter scale, the same principle of strain amplification applies to auxetic patterns with dimensions ranging from tens to hundreds of micrometers. For example, we design a photonic auxetic pattern containing arrays of three‐pointed stars with a width of 100 µm and a length of 280 µm using the photolithography technique as shown in Figure S13 (Supporting Information), which is potentially useful for colorimetric strain measurement at the millimeter scales. One of the promising applications of our ultra‐sensitive mechanochromic materials is the intuitive colorimetric monitoring of structural health for various architectures, as we demonstrate. There is potential to further develop the auxetic patterns to visualize strain fields, the distribution of the strain, and the deformation direction, which would increase the possible areas of application.

## 4 Experimental Section

### Materials

Monodisperse silica particles with an average diameter of 228 nm (Sukgyung AT) were homogeneously dispersed in ethanol. PEGPEA (Mn 324 g mol^−1^, Sigma Aldrich) containing 1 w/w% photoinitiator of 2‐hydroxy‐2‐methyl‐1‐phenyl‐1‐propaneone (Darocur 1173, Ciba chemical) was added into the ethanolic dispersion to have the volume fraction of 0.40 in an ethanol‐free basis. The ethanol was selectively evaporated from the dispersion in a convection oven at 70 °C for 12 h. To suppress the random scattering, carbon black nanoparticles were additionally dispersed in the ink at the concentration of 0.03 w/w%.

### Preparation of Auxetic Molds and Photonic Patterns

Master molds with negative auxetic patterns were prepared by a stereolithographic 3D printing (Form 3, Formlabs) with a photocurable resin (Grey resin, Formlabs). The molds were duplicated to have the same negative patterns with PDMS. The mixture of PDMS precursor (SYLGARD 184 silicone elastomer base, Dow) and curing agent (SYLGARD 184 silicone elastomer curing agent, Dow) in a 10:1 weight ratio was poured onto the master molds whose surface was coated with the mold release agent (Ease Release 200, Mann Release Technologies) and cured in an oven at 70 °C for 4 h. After releasing the PDMS from the master mold, the surfaces were treated by trichloro(1H,1H,2H,2H‐perfluorooctyl)silane through vapor deposition under vacuum for 1 h. The mixture of PDMS precursor and curing agent was poured onto the surface‐treated PDMS mold, which was then cured and released. The final PDMS molds with the same negative designs as the master molds were used as a new mold to make the framework of the auxetic pattern. A mixture of urethane acrylate (Miramer PU2100, Miwon Chemicals) and isobornyl acrylate (Mw 208.30 g mol^−1^, Sigma Aldrich) in a weight ratio of 3:1 containing 1 w/w% photoinitiator of 2‐hydroxy‐2‐methyl‐1‐phenyl‐1‐propanone (Darocur 1173, Ciba Chemical) was poured onto the PDMS mold and bladed with a slide glass. After photocuring of the acrylate mixture with UV exposure (CoolWave UV Curing System, Nordson) for 10 min, the auxetic patterns with void cuts were released from the PDMS mold. The void cuts were filled with the silica‐PEGPEA dispersions by sandwiching the auxetic patterns with a pair of glass slides in the presence of the 1.5 mL silica‐PEGPEA dispersions. After thermal annealing at 50 °C for 15 min, the patterns were exposed to UV for 1 min and then released from the glass slides.

### Characterization

The photonic patterns were observed by optical microscopy in a reflection mode (Eclipse L150, Nikon) and a stereo microscope (SMZ745T, Nikon). The reflectance spectra were measured using a fiber‐coupled spectrometer (USB 4000, Ocean Optics Inc.) equipped with optical microscopy, where a 20× objective lens with a numerical aperture of 0.45 and a field stop were used to measure the spectra from local areas. The mechanical properties were analyzed using a universal testing machine (MCT‐1150, AND Inc.).

### Statistical Analyses

In Figure 5d,g, data points on the graphs are represented as mean ± standard deviation. Three data points are used for each line. Statistical analysis was performed using OriginPro software (2019 version, Origin Lab). To analyze the quantitative relationship between hue and stain, the data were fitted to a bidose‐response function using MATLAB software (2023 version, MathWorks).

## Conflict of Interest

The authors declare no conflict of interest.

## Supporting information

Supporting Information

Supplemental Video 1

Supplemental Video 2

Supplemental Video 3

Supplemental Video 4

Supplemental Video 5

Supplemental Video 6

## Data Availability

The data that support the findings of this study are available in the supplementary material of this article.
