# Supplementary material for: Auxetic Photonic Patterns with Ultrasensitive Mechanochromism
Source: Adv Sci (Weinh). 2023 Nov 9;11(1):2304022. doi: 10.1002/advs.202304022 (PMC10767460; doi:10.1002/advs.202304022)
Supplement: Supplementary file 1 — Supporting Information [file ADVS-11-2304022-s007.pdf]

ADVANCED SCIENCE
Open Access

Supporting Information

for *Adv. Sci.*, DOI 10.1002/advs.202304022

Auxetic Photonic Patterns with Ultrasensitive Mechanochromism

Hwan-Young Lee, Minbon Gu, Jeonghee Hwang, Hyerim Hwang, Young-Seok Kim, Su Yeon Lee and Shin-Hyun Kim**

Supporting Information

Auxetic Photonic Patterns with Ultrasensitive Mechanochromism

Hwan-Young Lee,[1] Minbon Gu,[2] Jeonghee Hwang,[2] Hyerim Hwang,[3] Young-Seok Kim,[4] Su Yeon Lee,[2] and Shin-Hyun Kim[1,]*

[1]Department of Chemical and Biomolecular Engineering, Korea Advanced Institute of Science and Technology (KAIST)

[2]Division of Advanced Materials, Korea Research Institute of Chemical Technology (KRICT)

[3]Department of Chemical Engineering and Materials Science, Ewha Womans University

[4]Korea Electronics Technology Institute (KETI)

E-mail: Shin-Hyun Kim (kim.sh@kaist.ac.kr) and Su Yeon Lee (sylee@krict.re.kr)

Contents

S1. Sensitivity of mechanochromic materials in literature
S2. Fabrication of auxetic framework
S3. Reflectance spectrum of strain-free elastic photonic crystals
S4. Mechanochromism of bulk photonic film
S5. Influence of framework materials
S6. Reflectance spectra from local regions in double-cross
S7. Amplification of local strain in auxetic framework
S8. Consistency of short rectangular cuts in reflectance spectrum and color
S9. Reflectance spectra from local regions in horizontal line
S10. Reversibility of the auxetic photonic patterns
S11. Extension of auxetic photonic pattern until failure
S12. Extension of auxetic photonic pattern with narrower cuts until failure
S13. Extraction of the hue values for various strains
S14. High resolution auxetic patterns
S15. Description for Supporting Movies

S1. Sensitivity of mechanochromic materials in literature

The degree of lattice deformation directly influences the diffraction wavelength shift. In the case of compression, the peak shift can be calculated as the product of the initial peak wavelength and the strain, represented as $\Delta\lambda/\lambda_0 = \varepsilon$. The maximum sensitivity for this is $\lambda_0/100$ nm/%. Hence, the sensitivity cannot surpass 6.5 nm/% when λ_0 = 650 nm. For tensile strain, the maximum strain along the thickness direction is realized in purely elastic materials with a Poisson ratio of 0.5. Under these conditions, $\Delta\lambda/\lambda_0 = 0.5\varepsilon_x$, and the highest theoretical sensitivity is $\lambda_0/200$ nm/%. As such, the utmost sensitivity is 3.25 nm/% for λ_0 = 650 nm. In Table S1, we summarize the sensitivity of mechanochromic materials previously reported.

Table S1. Comparison of sensitivity

No.	Type	λ_o (nm)	$\Delta\lambda_{max}$ (nm)	Sensitivity (nm/%)	Reference
**1**	**Compressing**	640	300	6	*Nat. Commun.* **2014**, *5*, 4659.
**2**		680	250	4.16	*ACS Nano* **2023**, *17*, 5921.
**3**		640	233	4.7	*RSC Adv.* **2017**, *7*, 33258.
**4**		525	58	2.63	*Adv. Funct. Mater.* **2014**, *24*, 3197.
**5**		530	55	2.3	*ACS Nano* **2021**, *15*, 8770.
**6**	**Stretching**	641	111	2.77	*Adv. Funct. Mater.* **2023**, *33*, 2213099.
**7**		660	110	2.75	*Adv. Mater.* **2019**, *31*, 1805496.
**8**		620	135	1.96	*ACS Nano* **2017**, *11*, 11350.
**9**		610	190	1.72	*Chem. Mater.* **2019**, *31*, 8918.
**10**		620	150	0.73	*Nat. Mater.* **2022**, *21*, 1441.

S2. Fabrication of auxetic framework

The auxetic framework is fabricated by molding. A master mold with a positive pattern is prepared by 3D printing as shown in Figure S1a. The master mold is replicated to have the same design with PDMS, as shown in Figure S1b. A mixture of urethane acrylate and isobornyl acrylate in a weight ratio of 3:1 containing 1 w/w% photoinitiator is poured on the PDMS mold and cured by UV irradiation. The polymerized framework is released from the mold, as shown in Figure S1c.

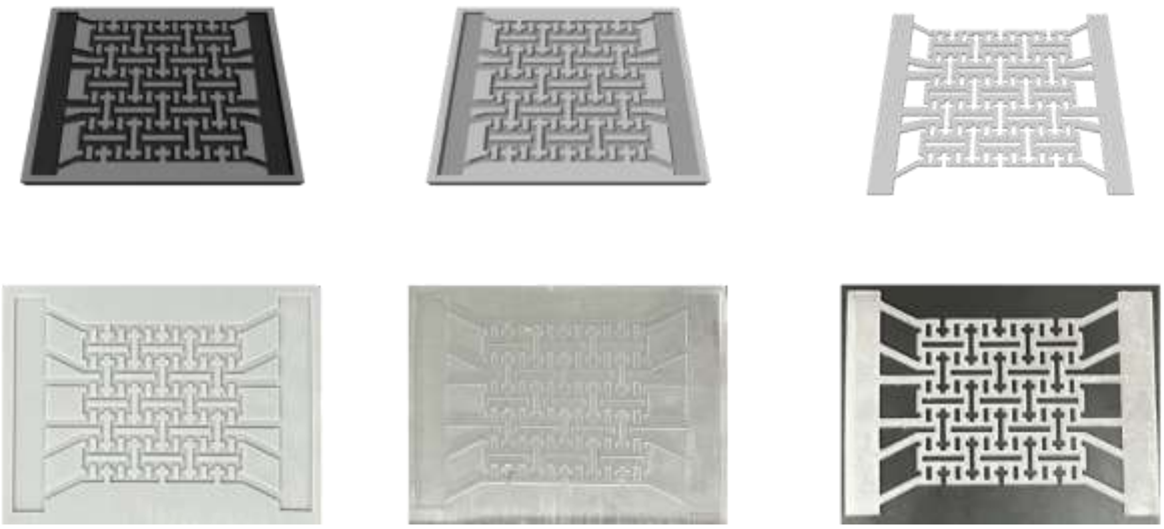

Figure S1. (a-c) Sets of schematic and photograph of master mold prepared by 3D printing (a), PDMS mold replicated from the master mold (b), and auxetic framework molded from the PDMS mold (c).

S3. Reflectance spectrum of strain-free elastic photonic crystals

The regular arrays of silica particles in polymerized PEGPEA makes a reflection peak in the spectrum through Bragg diffraction. For example, the arrays of silica particles with an average diameter of 228 nm at the volume fraction of 0.40 show a peak at the wavelength of 676 nm, as shown in Figure S2.

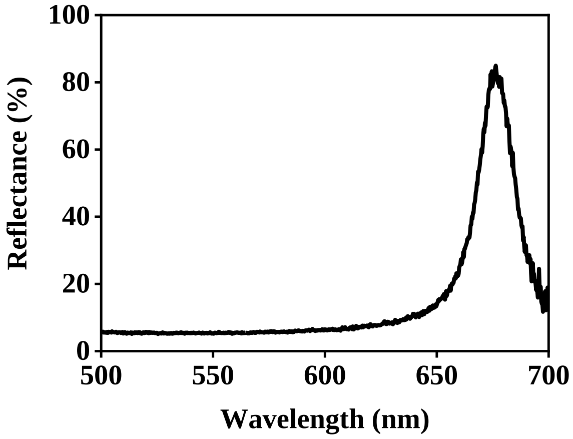

Figure S2. Reflectance spectrum of elastic photonic crystals composed of non-close-packed arrays of silica particles in polymerized PEGPEA matrix.

S4. Mechanochromism of bulk photonic film

The elastic photonic film shows strain-dependent reversible color change due to the structural deformation of colloidal arrays in the film. As the strain increases from 0, the film turns from red to orange at 10% strain, green at 30%, and cyan at 50%, as shown in Figure S3a. The reflectance peak also blueshifts along with the strain, as shown in Figure S3b. The peak position is almost linear to the strain up to almost 50% strain at which the average slope is 2.9 nm/%, as shown in Figure S3c.

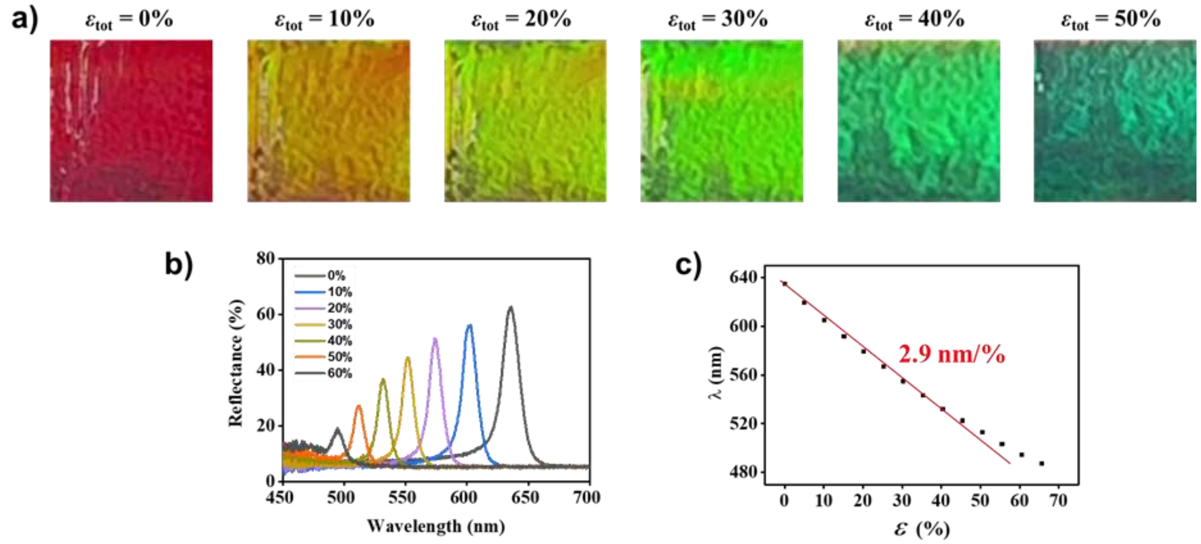

Figure S3. (a) Series of photographs of bulk elastic photonic film showing the gradual blueshift of color from red at strain-free state to cyan at 50% strain. (b) Reflectance spectra of bulk film taken at various strains as denoted. (c) Peak position as a function of strain. There is a linear relation in the range of strain from 0 to 50%, where an average slope is 2.9 nm/%.

S5. Influence of framework materials

When urethane acrylate is used as a framework material without isobornyl acrylate, the modulus is relatively low and the blueshift of the photonic crystal in the cut area is relatively limited, as shown in Figure S4a. The use of the mixture of urethane acrylate and isobornyl acrylate in a weight ratio of 3:1 increases the modulus of the framework and provides enhanced degree of blueshift, as shown in Figure S4b. It seems that large modulus contrast between the framework and elastic photonic crystals enables the higher degree of strain localization in the cut areas.

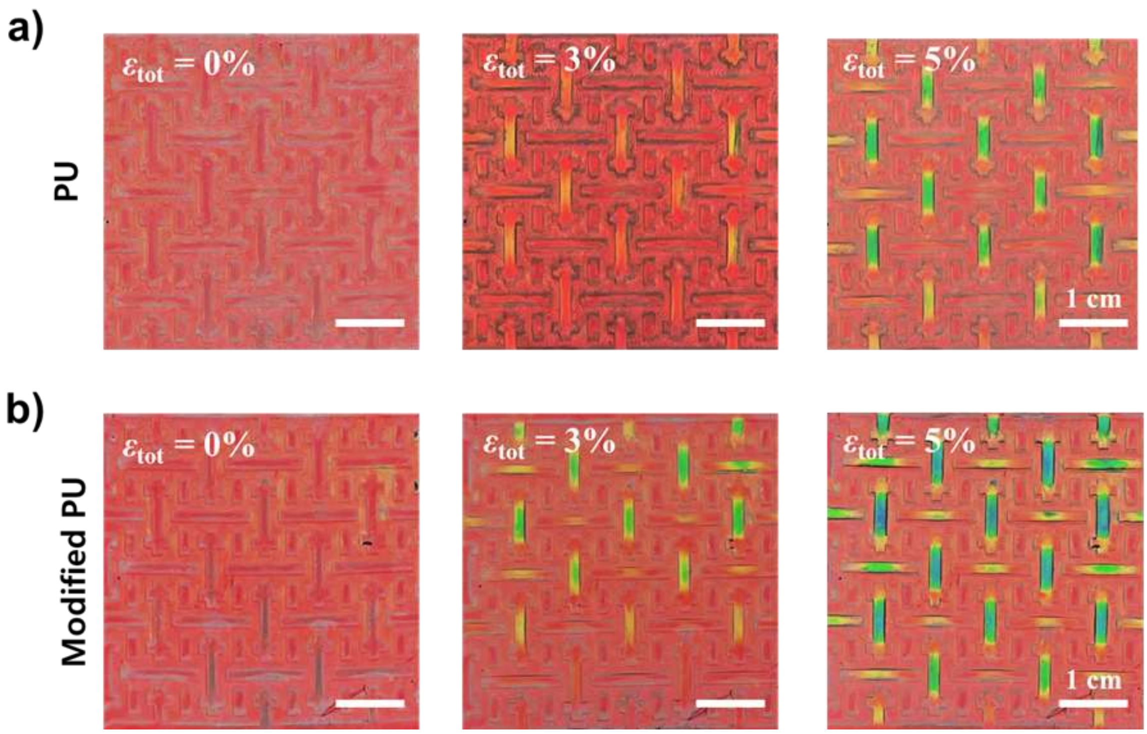

Figure S4. (a,b) Series of photographs of auxetic photonic patterns of which framework is made of polyurethane (a) and poly(urethane-co-isobornyl) (b).

S6. Reflectance spectra from local regions in double-cross

There is a regional variation of the degree of blueshift within a single cut element of double-crosses. To study the variation quantitatively, the reflectance spectra are acquired from five different locations, as indicated in Figure S5a. The spectra at five locations are measured for various strains of 0, 1, 2, 3, 4, and 5%, as shown in Figure S5b-g. The degree of blueshift increases along with the strain, where the central region (#1) shows greatest shift.

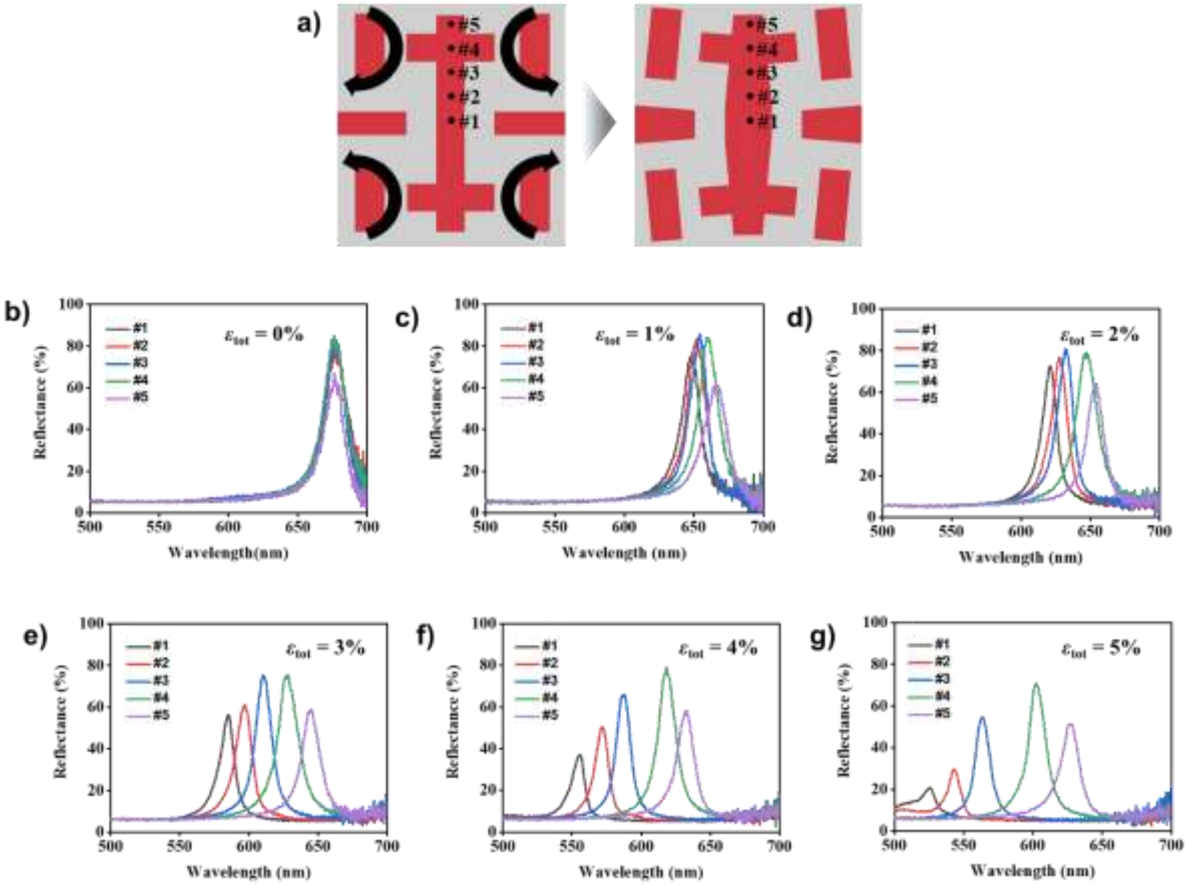

Figure S5. (a) Cartoons showing the deformation of the sub-element, double-cross, where five different locations are denoted with #1-#5. (b-g) Reflectance spectra taken at the five locations at the strain-free state (b), 1% strain (c), 2% (d), 3% (e), 4% (f), and 5% (g).

S7. Amplification of local strain in auxetic framework

The auxetic framework amplifies strain in the cut areas. To compare the mechanical response of the framework with auxetic pattern containing photonic crystal, the framework is monitored during the expansion and the local strains in the double-cross and horizontal line are measured from image analysis, as shown in Figure S6. There is no significant difference in the local strains between the photonic crystal-free framework and the photonic crystal-embedded pattern, as shown in Figure S6c.

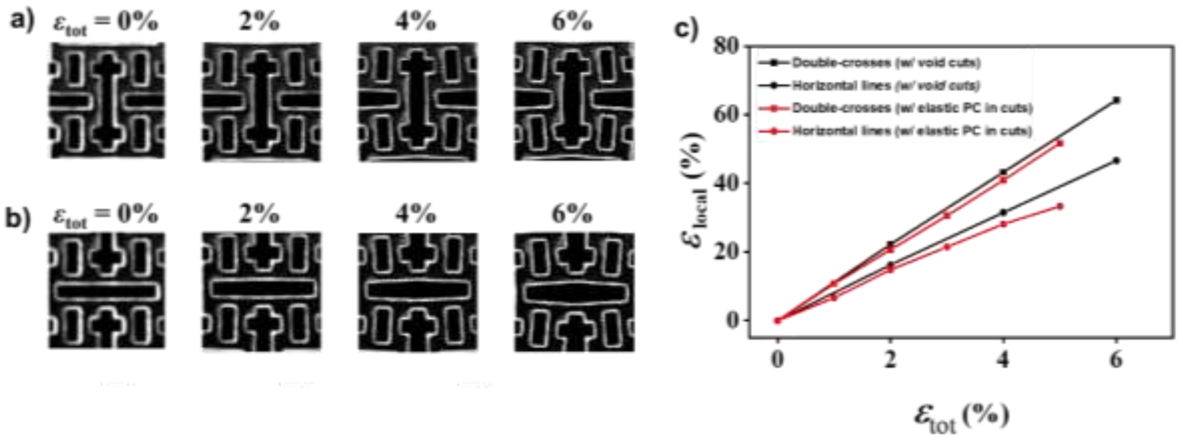

Figure S6. (a,b) Series of OM images showing the gradual deformation of double-cross (a) and horizontal line (b) in the auxetic framework along with strain. (c) Local strains at the centers of double-cross and horizontal line as a function of total strain for auxetic framework and photonic patterns.

S8. Consistency of short rectangular cuts in reflectance spectrum and color

To study the consistent color preservation of the short rectangular cuts, we measure the reflectance spectra and optical microscope (OM) images under various strains, as shown in Figure S7. Notably, no changes are observed in the reflectance spectrum and color of the short rectangular cuts. This characteristic makes them a valuable reference for quantifying color changes during strain analysis.

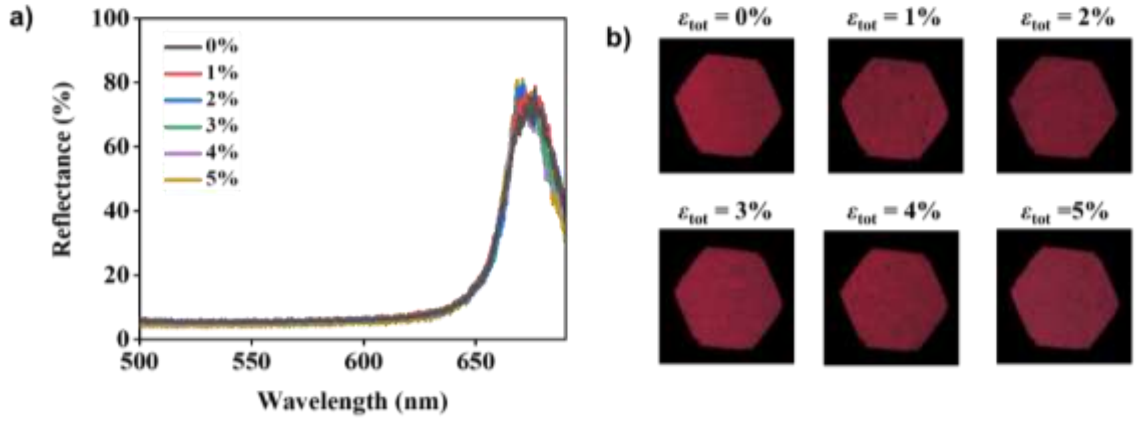

Figure S7. (a, b) Reflectance spectra (a) and OM images (b) of the short rectangular cut for various strains as denoted.

S9. Reflectance spectra from local regions in horizontal line

There is a regional variation of the degree of blueshift within a single cut element of horizontal line. To study the variation quantitatively, the reflectance spectra are acquired from three different locations, as indicated in Figure S7a. The spectra at three locations are measured for various strains of 0, 1, 2, 3, 4, and 5%, as shown in Figure S7b-g. The degree of blueshift increases along with the strain, where the central region (#a) shows greatest shift.

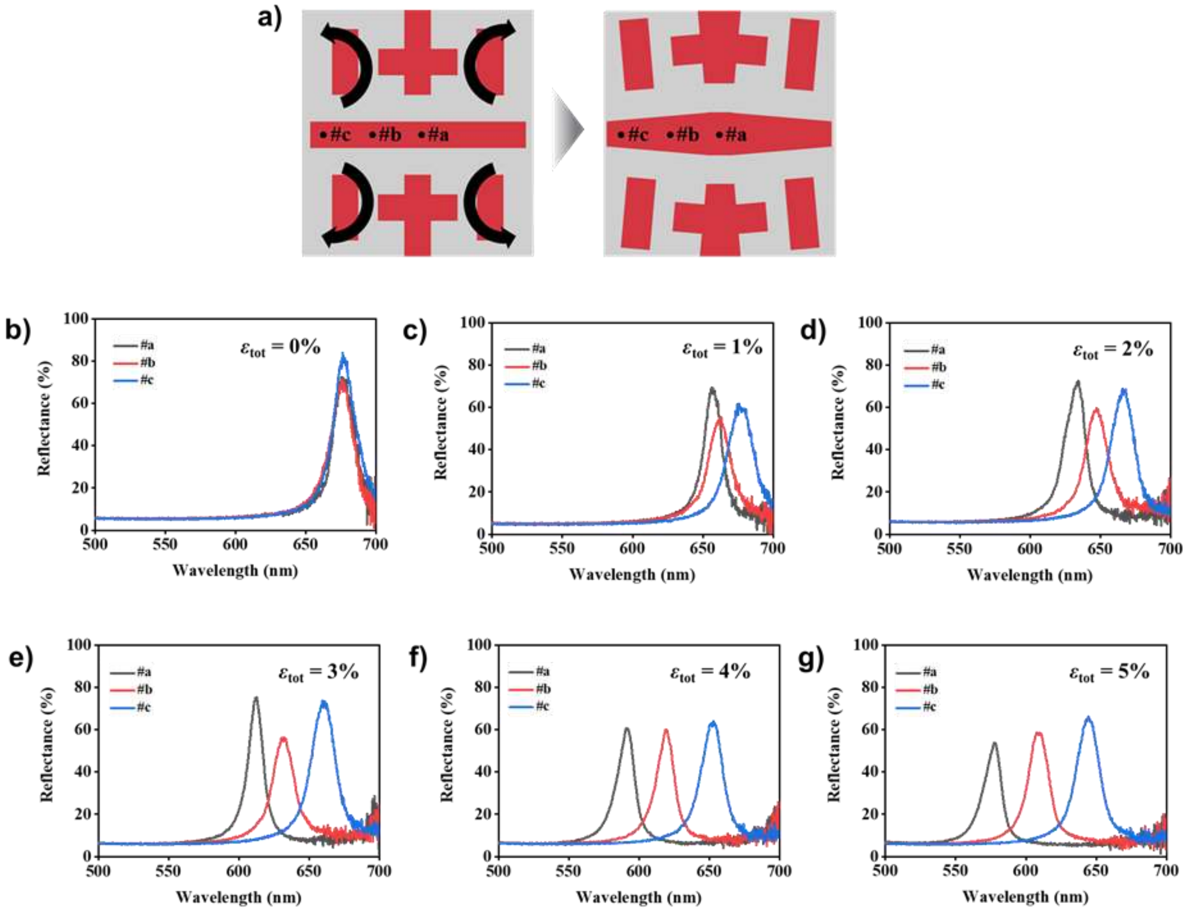

Figure S8. (a) Cartoons showing the deformation of the sub-element, horizontal line, where three different locations are denoted with #a-#c. (b-g) Reflectance spectra taken at the three locations at the strain-free state (b), 1% strain (c), 2% (d), 3% (e), 4% (f), and 5% (g).

S10. Reversibility of the auxetic photonic patterns

To investigate the fatigue durability of the auxetic photonic patterns, we conduct cycling tests, as shown in Figure S9. Throughout 310 cycles between the total strains of 0 and 3%, no discernible change in the response is observed. This demonstrates the robustness and high

stability of the patterns under repeated deformation. Furthermore, we observe the color change and recovery are instantaneous, occurring without any noticeable delay.

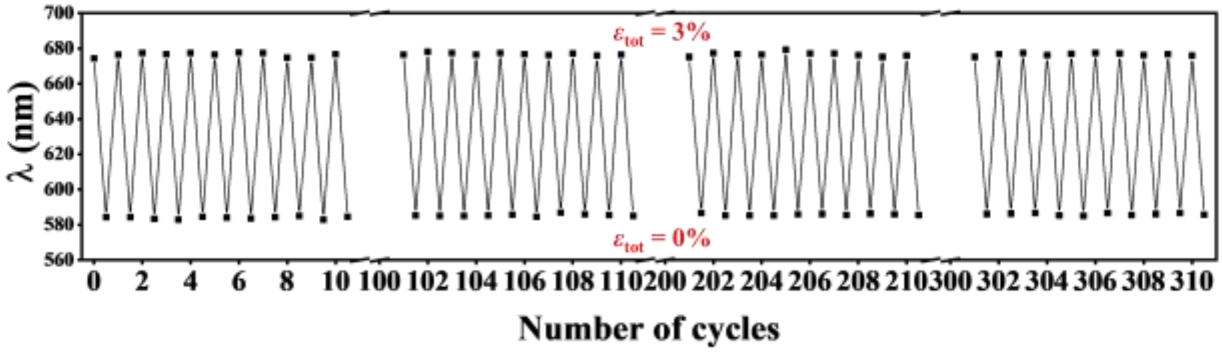

Figure S9. Reversible change of reflectance peak position during 310 cycles of extension to 3% and relaxation to 0%.

S11. Extension of auxetic photonic pattern until failure

The auxetic photonic patterns exhibit remarkable stability, with no plastic deformation or failure observed up to a maximum strain of 12%. The failure occurs at the interfaces between photonic material and auxetic framework at 13%, which gets worse along with the strain, shown in Figure S10. This high stability is achieved by producing a thin film of photonic materials that envelops the entire auxetic pattern. However, when the total strain exceeds 5%, a nonlinear response occurs due to the inherent limitations of the photonic materials.

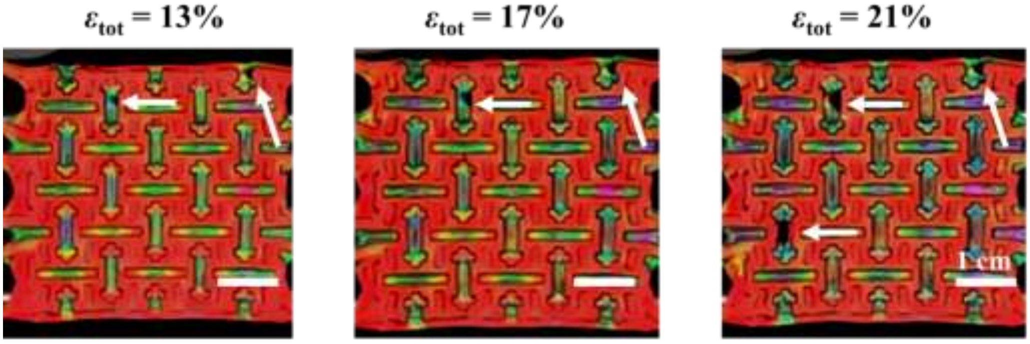

Figure S10. Series of photographs of an auxetic photonic pattern with a width of 1.4 mm at three different strains, as denoted.

S12. Extension of auxetic photonic pattern with narrower cuts until failure

The auxetic photonic patterns with narrow cuts of 0.7 mm offer higher sensitivity compared to patterns with wide cuts of 1.4 mm. However, the patterns with narrow cuts exhibit a more restricted colorimetric measurement range for strain analysis and early failure, as shown in S11. Reliable measurement range is 0 - 2%, where a linear response is preserved. The failure occurs at the strain of 5%.

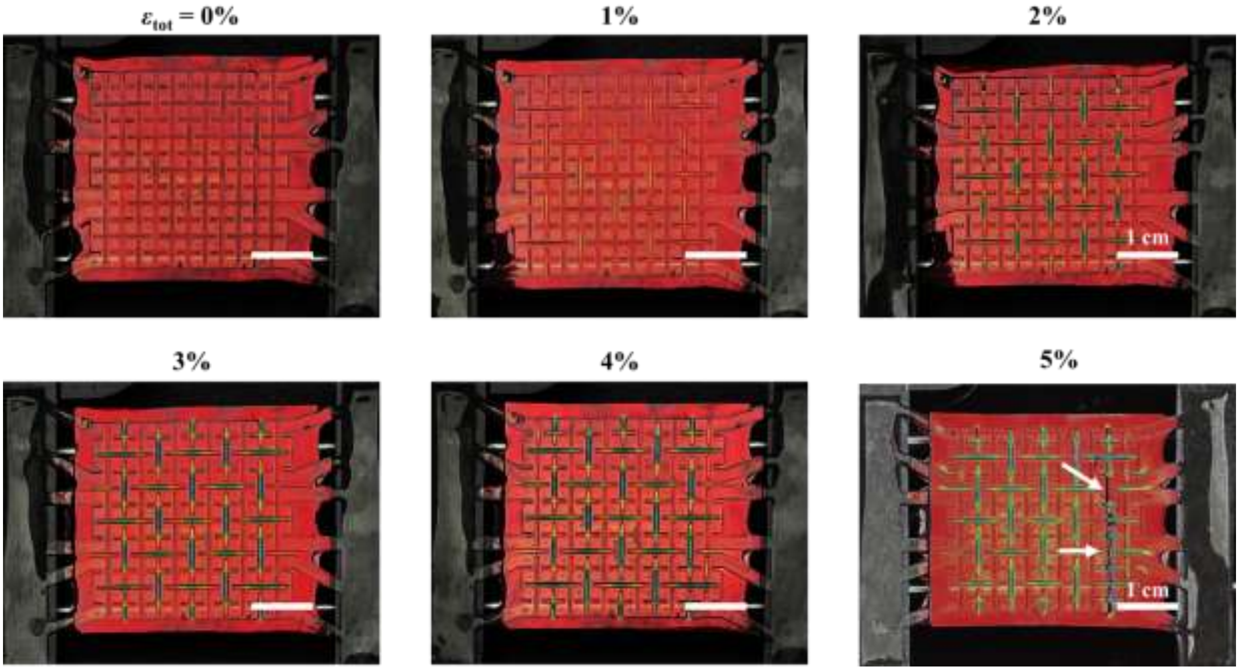

Figure S11. Series of photographs of an auxetic photonic pattern with a width of 0.7 mm at three different strains, as denoted.

S13. Extraction of the hue values for various strains

To obtain a standard curve for the relationship between strain and hue values, we take images of the auxetic pattern for various strains in the range of 0 - 4%, as shown in Figure S8. The hue values are extracted from the central regions of the double-cross and horizontal line in the image and averaged for the same cut elements, as summarized in the right panel of Figure 5a.

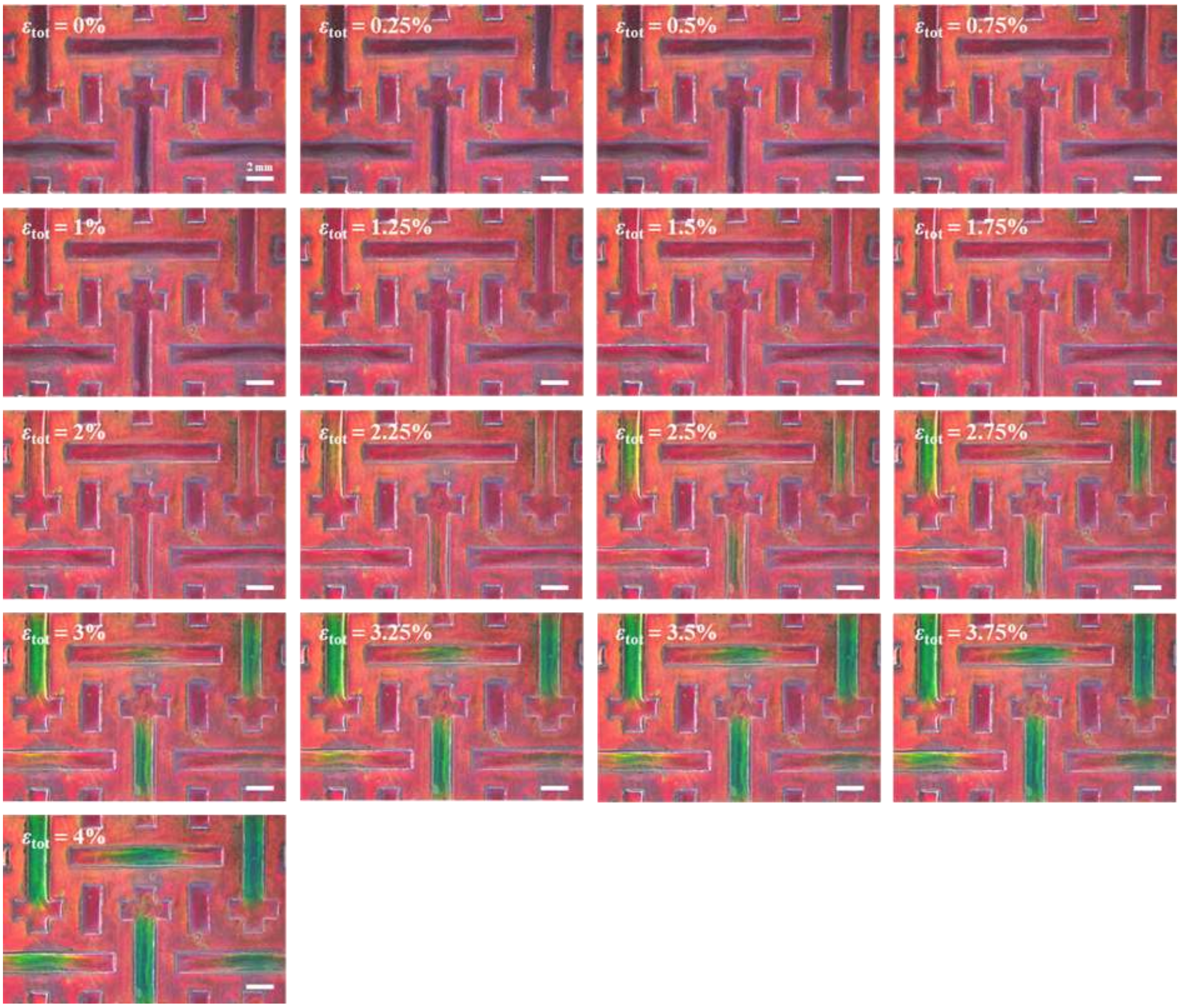

Figure S12. Series of OM images of the auxetic photonic pattern for various strains, as denoted.

S14. High resolution auxetic patterns

We can prepare high resolution auxetic patterns through photolithography and soft lithography. For example, the mold for the auxetic pattern composed of arrays of three-pointed stars is prepared to have a line width of 100 μm and a length of 280 μm by photolithography technique, with which the auxetic pattern with void cuts is replicated by soft lithography using modified PU, as shown in Figure S13a. By following the same protocol for the macropatterns, we can produce photonic auxetic patterns with high resolution, as shown in Figure S13b.

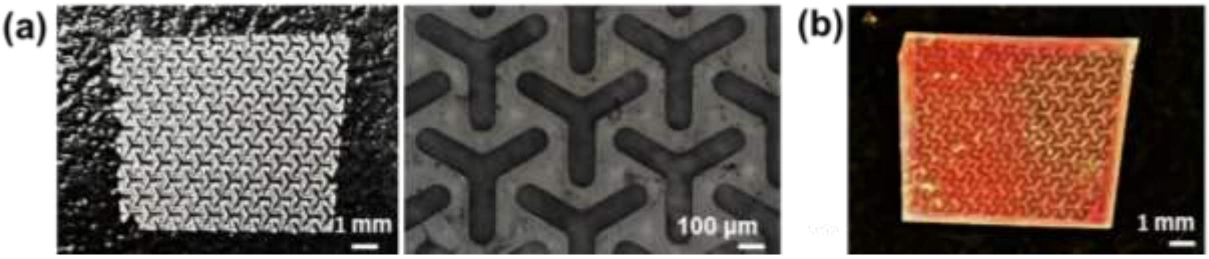

Figure S13. (a) Photograph and OM image of the auxetic pattern with void cuts produced by photolithography and soft lithography.

S15. Description for Supporting Movies

- **Movie S1**: Mechanochromism in auxetic photonic pattern with a cut width of 1.4 mm.
- **Movie S2**: Reversible color change of auxetic photonic pattern during stretching and relaxation.
- **Movie S3**: Mechanochromism in bulk photonic film.
- **Movie S4**: Color change in double-cross of auxetic photonic pattern.
- **Movie S5**: Color change in horizontal line of auxetic photonic pattern.
- **Movie S6**: Reversible mechanochromism in auxetic photonic pattern with a cut width of 0.7 mm.
